# Preventive treatment options for fear of falling within the Swiss healthcare system: A position paper

**DOI:** 10.1007/s00391-021-01957-w

**Published:** 2021-09-29

**Authors:** Eric Lenouvel, Lan Novak, Andreas Biedermann, Reto W. Kressig, Stefan Klöppel

**Affiliations:** 1 University Hospital of Old Age Psychiatry and Psychotherapy, University of Bern, Murtenstraße 21, 3008 Bern, Switzerland; 2 Graduate School for Health Sciences, University of Bern, Bern, Switzerland; 3 Public Health Services, Bern, Switzerland; 4 University Department of Geriatric Medicine FELIX PLATTER & University of Basel, Basel, Switzerland

**Keywords:** A matter of balance, Balance confidence, Falls efficacy, Cognitive behavioral therapy, A matter of balance, Gleichgewichtsvertrauen, Sturzprävention, Kognitive Verhaltenstherapie

## Abstract

Fear of falling (FoF) results in social, functional, physical, and psychological symptoms, including secondary disorders, such as depression and general anxiety disorder (GAD). A vicious cycle develops, where symptoms maintain and reinforce FoF and its consequences, including increasing the risk of falling. In this position paper, we suggest screening for FoF using the falls efficacy scale international (FES-I) questionnaire. The presence of a high score (> 23) warrants an investigation into frailty and exclusion of depression and GAD, during the clinical interview. Stratifying frailty, based on the Fried frailty criteria will guide treatment options based on the most significant health concerns. Frail older adults should first receive physiotherapy and exercise interventions, as physical disabilities are their most significant characteristic, while pre-frail and non-frail older adults should receive multicomponent interventions, consisting of cognitive behavioral therapy (CBT) with physical exercise. The non-frail with predominantly GAD and depression should receive specialized CBT interventions. Currently, only exercise interventions are available for FoF treatment in Switzerland. Although some exercise interventions use CBT elements, such as goal setting and reflections on behavior and feelings, they are not systematically used, are not part of a quality-assured procedure, and do not address the psychological-cognitive aspects of FoF. As the pre-frail and non-frail are the largest groups to use these services, adapting current exercise programs by incorporating a CBT component would be the most practical means to provide optimized care.

## Abstract (de)

Sturzangst führt zu sozialen, funktionellen, körperlichen und psychischen Symptomen, einschließlich sekundärer Störungen, wie Depression und generalisierte Angststörung (GAS). Es entsteht ein Teufelskreis, bei dem die Symptome die Sturzangst und ihre Folgen, einschließlich der Erhöhung des Sturzrisikos, aufrechterhalten und verstärken. In diesem Positionspapier schlagen wir vor, ein Screening auf Sturzangst mit dem Fragebogen Falls Efficacy Scale International (FES-I) durchzuführen. Das Vorhandensein eines hohen Wertes (> 23) rechtfertigt eine Untersuchung auf Gebrechlichkeit und erfordert den Ausschluss von Depression und GAS im Rahmen eines klinischen Interviews. Die Stratifizierung der Gebrechlichkeit, basierend auf den Fried-Kriterien für Gebrechlichkeit, leitet die Behandlungsoptionen basierend auf den wichtigsten Gesundheitsproblemen. Gebrechliche ältere Erwachsene sollten zuerst Physiotherapie und Bewegungsinterventionen erhalten, da körperliche Einschränkungen ihr wichtigstes Merkmal sind, während nicht gebrechliche ältere Erwachsene und solche mit Vorstufen von Gebrechlichkeit eine Multikomponenten-Intervention erhalten sollten, bestehend aus kognitiver Verhaltenstherapie (CBT) mit körperlicher Bewegung. Nicht gebrechliche Menschen mit überwiegend GAD und Depressionen sollten spezielle CBT-Interventionen erhalten. Derzeit sind in der Schweiz nur Bewegungsinterventionen für die Behandlung von Sturzangst verfügbar. Obwohl einige Übungsinterventionen CBT-Elemente verwenden, wie z. B. Zielsetzung und Reflexion über Verhalten und Gefühle, werden diese nicht systematisch eingesetzt, sind nicht Teil eines qualitätsgesicherten Verfahrens und gehen nicht auf die psychologisch-kognitiven Aspekte von Sturzangst ein. Da Personen ohne bzw. mit Vorstufen von Gebrechlichkeit die größten Gruppen darstellen, die diese Dienste in Anspruch nehmen, wäre die Anpassung aktueller Trainingsprogramme durch Einbindung einer CBT-Komponente das praktischste Mittel, um eine optimierte Versorgung zu gewährleisten.

## Background

Fear of falling (FoF) is a “lasting concern about falling that leads to an individual avoiding activities that he/she remains capable of performing [32].“ It is considered an entity of falls-related psychological concerns, which also includes falls efficacy, which describes how a person perceives their ability not to fall [24]. The FoF, depending on the number of previous falls, is present in up to 86% of older adults (65 years and over) [36]. Additionally, there is increased prevalence of FoF among those suffering from mild to moderate cognitive impairment and among the frail [21, 23, 29]. To put these figures into perspective, up to 35% of older adults will fall each year, increasing in frequency with age and frailty [34].

The FoF has been suggested to be unidirectionally linked to falls efficacy and anxiety, in that high levels of FoF lead to low levels of falls efficacy, resulting in a maladaptive nature of FoF, where daily activities appear to have excessive risk for falling [1]. High levels of FoF result in social, functional, physical, and psychological symptoms, such as avoidance, decrease in social participation, ability to perform activities of daily living (ADLs), quality of life, and self-confidence, increased rates of depression and general anxiety [29, 36]. These various sequelae have been shown to develop into a vicious cycle, where symptoms maintain and reinforce FoF, and its consequences, including increasing the risk of falling [9]; however, low levels of FoF have been suggested by some authors to even protect against future falls [1].

Frailty is a condition of decreased functional reserve leading to a vulnerable state and is considered the stage before disability [18]. Due to the expanding old age population throughout the world, frailty is an emerging global health challenge. The levels of frailty among older adults, and as such their treatment needs, are highly heterogeneous [8, 18]. Although different models of frailty exist with no current consensus, many describe a continuum between healthy and frail, such as the functional spectrum by Speechley and Tinetti, 1991, or the more recent Fried frailty criteria, commonly used in studies of older adults [10, 20]. The Fried criteria group older adults into non-frail, pre-frail, and frail. Frailty occurs when three or more criteria are met of weakness, slow gait, low physical activity, exhaustion, and unintentional weight loss, whereas pre-frail means that one to two of these criteria are fulfilled [10].

**Fig. 1:**
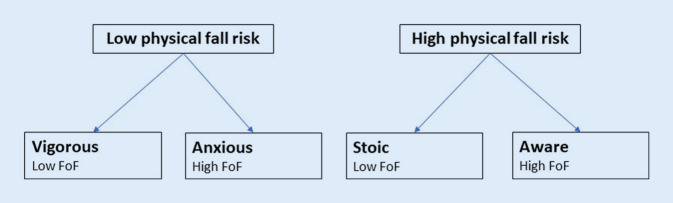
Simplified Delbaere classification tree, modified from Delbaere et al. [5]

The FoF is associated with frailty and various health characteristics [21]. Delbaere et al. classified older adults based on the disparity between the FoF level and physiological state; Vigorous—low FoF and low fall risk, anxious—high FoF and low fall risk, stoic—low FoF and high fall risk and aware—high FoF and high fall risk (*see* Fig. 1; [5]). These groups are found in 29%, 11%, 20%, and 40% of older adults living in the community, respectively, each having a rate of one or more falls during a 1-year period of 20%, 39%, 34%, and 41%, respectively [5]. The largest group with FoF, the aware, have physical limitations leading to high fall risk that would likely classify them as at least pre-frail older adults according to the Fried criteria. Older adults are expected to progress through the frailty spectrum.

Although FoF is associated with geriatric multimorbidity and immobility, FoF is also associated with both anxiety and depression [5, 7, 26]. The FoF has been associated with a wide range of nonfall-related concerns, such as being robbed, forgetting appointments, having financial problems, losing one’s personal identity or independence [13, 14, 35]. This wide range of concerns resembles the excessive, difficult to control, and disturbing anxiety found in general anxiety disorder (GAD). Late life anxiety might present as FoF, whereas it may have presented with other symptoms in earlier phases of life. A study by Payette et al. even suggested that FoF has a greater association to GAD than to actual fall risk [26]. The association of FoF with depression has also been demonstrated in several studies, although it is not clear whether depression is the consequence of FoF or vice versa [7, 11, 15, 16]. As a consequence, and because GAD and depression can be the cause of FoF at least in some patients, screening, diagnosing, and possibly treating primary psychiatric disorders should be undertaken [28].

Often overlooked in favor of somatic causes of reduced mobility and increased frailty, FoF identification and treatment is important, due to its prevalence and the symptoms it can cause even among those who have not fallen, to prevent and treat the physical and psychological sequalae [29]. Despite this, there is no generally accepted consensus on screening and treating FoF. Sufficient studies are lacking to synthesize evidence for best screening and treatment options in Switzerland. In this position paper, we suggest that screening for FoF using the falls efficacy scale international (FES-I) questionnaire, as well as screening for depression and GAD in the clinical interview, while stratifying frailty, based on the Fried Frailty criteria, will help identify FoF and guide treatment options based on core needs. This position paper is based on significant contributions from scientific literature and consensus opinion among the multidisciplinary group of expert coauthors.

## Treatment options for FoF

The FoF is an interdisciplinary problem. Geriatricians and family physicians, in being primary care practitioners, typically manage the somatic sequelae, falls, and immobility resulting from FoF. Psychiatrists respond to the fear and anxiety response to the thought of falling, and the resulting vicious cycles leading to psychiatric comorbidities. As such, treatment options should address both the somatic and psychiatric needs of individuals, while focusing on their greatest needs. Cognitive behavioral therapy (CBT), multicomponent (CBT combined with exercise), and exercise interventions have been developed to treat FoF [4, 19, 22]. CBT is a form of psychotherapy that seeks to modify dysfunctional beliefs and behavior through various psychotherapeutic techniques [22]. Exercise interventions are planned, structured, repetitive, and purposive physical activities aimed at improving or maintaining one or more components of physical fitness [19]. Multicomponent interventions incorporate both CBT and exercise components into the interventions. Treatment of FoF aims to reduce the symptoms of FoF and reduce the associated fall risk factors [4]. These interventions should aim to decrease the number of falls, increase strength, gait, balance and mood, give a better ability to get up after a fall, and have an increased number of activities performed without falling [19, 30].

Parry et al. developed a purely CBT intervention for FoF, given individually at home over 45 min on a weekly basis for 8 weeks with a follow-up session at 6 months, by specially trained healthcare assistants. A CBT intervention with an exercise component for FoF was first developed by Tennstedt et al. and adapted in different countries [4, 22, 25, 31]. Tennstedt’s intervention uses three levels of facilitators; a coach, who gives the class, receives 8 h of training. They are trained by master trainers, who have completed 2 full days of training by lead trainers, who are specialists in the field. This structure permits a cost-effective solution. This demonstrates how inexperienced therapists can be just as effective as expert therapists when a treatment plan is followed [33]. Exercise interventions such as Tai Chi, balance training or dancing likely increases physical capabilities and improves confidence to perform activities [19]. They have been shown to reduce FoF without increasing the risk of falls. Sessions are given by experts one or more times per week, for 10–13 weeks in many protocols. In Switzerland, Tai chi coaches receive certification from Tai Chi schools after following regular training over two semesters and after a final examination. Meta-analyses have shown that the effect sizes of psychotherapy, exercise interventions, and their combination in multicomponent interventions overlap [4, 19, 22]. This effect is most commonly measured using the Falls Efficacy Scale Internation (FES‑I), which screens for the level of concern regarding falling that an individual may have [19, 32]. A score of > 23 for the 16-item FES‑I scale corresponds to a high concern for falling, equating to a high FoF [6].

There is insufficient evidence to tailor specific FoF interventions based on the level of frailty of an individual with FoF. Current best practice would therefore target treatment towards their most significant health concern [8, 21, 22]. Using the Fried criteria therefore should optimize treatment and focuses the intervention to address the greatest needs of the individual. Frail older adults primarily have physical disabilities that would lead to an increased fall risk, such as sarcopenia. It is therefore reasonable to first offer this group physiotherapy and exercise interventions to help restore or maintain strength and balance. Those in the pre-frail stage are in the process of losing their physical abilities, increasing risk of falls, regardless of the level of FoF. Multicomponent interventions should allow this group to best preserve their abilities. They may serve as an opportunity to address physical problems as well as address FoF’s vicious psychological cycles, through the CBT component [21]. Anxiety is likely the primary manifestation of FoF in non-frail older adults. They do not yet have the physical limitations of the frail and avoid activities that they remain physically capable of performing. A multicomponent intervention would reinforce and maintain current physical capabilities through its exercise component, and the CBT component would address this more significant anxiety component. Non-frail older adults presenting predominantly with anxiety or depression will likely benefit from CBT interventions, such as that developed by Parry et al.

## Context in Switzerland

In Switzerland, about 88,000 annual falls have been reported in people aged 65 years and over, and 1520 fall-related deaths per year were found between 2011 and 2015 [2]. The resultant health costs represent a significant economic burden, costing up to 1.5% of all healthcare expenditure [12]. In Switzerland, this should represent around 1.2 billion CHF [2].

Despite these costs and the prevalence of FoF, it is unlikely to be recognized. Physicians are pressed for time and other resources, with varying attitudes towards mental health, and with a risk to misinterpret the results of measurement tools [17, 27]. This may contribute towards barriers to participation. There may be denial or underestimation of the risk of falling, presence of FoF, fatalism (e.g. too old to start), social stigma, and inaccessible information [3]. Although there are no specific federal directives for preventive medicine in old age, several organizations have engaged in fall prevention, supported by both health insurers and cantonal efforts. In this respect organizations such as the *Bureau de Prévention des Accidents* (BPA), Promotion Santé Suisse, Pro Senectute, Physioswiss, the Swiss Association of Ergotherapists (ErgotherapeutInnen-Verband Schweiz), Rheumaliga Schweiz have either individually or in partnership, introduced programs like *Sicher gehen—Sicher stehen (Sichergehen.ch)*, and *StoppSturz* aimed at developing, improving quality, and providing fall prevention programs in the old age. Of particular note, *StoppSturz* is currently developing, as part of its fall prevention program, guidelines for the management of FoF. *StoppSturz* aims to help healthcare providers to recognize, clarify, prevent, and treat underlying causes of falls. In forming an interdisciplinary expert committee, they aim at providing the highest quality tools and training for healthcare providers. The steering committee is comprised of general practitioners, nurses, ergotherapists, physiotherapists, and geriatricians. The precise numbers of participants in each fall prevention program and total number of fall prevention groups are not publicly known. This is likely due to the fact that many exercise groups for seniors include balance and strength training but do not consider fall prevention their primary goal.

## Treatment options for FoF within the Swiss context

Currently it appears that there are few specific FoF interventions available in Switzerland. *SturzZentrum Schweiz*, based in Zurich, offers targeted evidence-based interventions for FoF, and training for coaches. Interventions offered are Tai Chi, Dalcroze eurhythmics training, dance match home-based programs, and strength and balance training. *Pro Senectute*, provides Tai Chi classes specific for FoF, and FitGym specific for fall prevention. The *Schweizerischer Turnverband *offers “*Turnen Erwachsene*” exercise classes. *Sichergehen.ch*, a comprehensive website listing various fall prevention activities for older adults throughout Switzerland, lists different evidence-based interventions for fall prevention. To date, *Sichergehen.ch* only has exercise interventions. Although some *Sichergehen.ch* exercise interventions use elements found in CBT, such as goal setting and reflections on behaviour and feelings, they are not systematically used, are not part of a quality assured procedure, and do not address the psychological cognitive aspects of FoF. CBT interventions or multicomponent interventions for FoF are not known to be given in Switzerland.

## Opportunities to develop a multicomponent intervention for the Swiss context

**Fig. 2:**
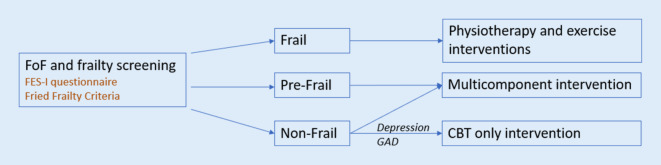
Treatment algorithm of FoF

Many patients with high levels of FoF using the fall prevention exercise programs would likely benefit from a multicomponent intervention. The adaptation of a CBT component to currently existing programs would likely be the most practical means of reaching the greatest number of individuals. Developing multicomponent interventions for FoF may incorporate validated FoF treatment models, such as Tennstedt et al.’s “A matter of balance©”, by adapting them to currently available exercise interventions given by the *Rheumaliga Schweiz, Pro Senectute*, or the *Schweizerischer Turnverband* [31]. This adapted multicomponent model would therefore correspond to the Swiss context. Primary healthcare providers who screen for FoF and frailty could then have more options on treating FoF and guiding their patients towards an optimized care, as shown in Fig. 2. Healthcare providers can administer the FES‑I questionnaires and determine the Fried frailty criteria. If the FES‑I questionnaire scores greater than 23, then based on the Fried frailty criteria, optimized treatment could be suggested; physiotherapy and exercise interventions for the frail, combined CBT and exercise interventions for the pre-frail and non-frail, and CBT interventions for the non-frail with primarily psychiatric symptoms of depression and anxiety, as identified during clinical interviewing. In order to address the fall prevention needs of the vigorous group, they can be encouraged to pursue currently available fall prevention programs. Additionally, as the fall prevention programs typically consist of exercise interventions, there is potential to develop a cognitive intervention component for these interventions, focusing on learning from previous or near falls.

## Conclusion

Fear of falling is a prevalent problem among older adults that creates a vicious cycle leading to the development of physical and mental comorbidities. Targeting treatment when there is a high concern for falling (FES-I > 23), based on the Fried frailty criteria will optimize treatment. Frail older adults should first receive physiotherapy and exercise interventions, as physical disabilities are their most significant characteristic, while pre-frail and non-frail older adults should receive multicomponent cognitive behavioral therapy (CBT) exercise interventions. The non-frail with predominantly anxiety and depressive symptoms should receive CBT interventions. Incorporating CBT elements in currently validated exercise interventions can establish multicomponent interventions specific for the Swiss context.
